# A Feature-Enhanced Deep Learning Network for GPR Hyperbolic Target Detection in B-Scan Profiles

**DOI:** 10.3390/s26185872

**Published:** 2026-09-16

**Authors:** Xin Wang, Qinghe Zhang, Han Liu

**Affiliations:** 1 College of Computer and Information Technology, China Three Gorges University, Yichang 443002, China; 202408100021119@ctgu.edu.cn (X.W.); liuh@ctgu.edu.cn (H.L.); 2 Hubei Key Laboratory of Intelligent Vision Based Monitoring for Hydroelectric Engineering, China Three Gorges University, Yichang 443002, China

**Keywords:** B-scan images, hyperbola detection, YOLOv8, lightweight multi-scale feature extraction

## Abstract

(1) Background: To address the challenges in ground-penetrating radar (GPR) B-scan profiles—where targets typically exhibit hyperbolic signatures, small scales, low contrast, and severe background clutter—this paper proposes an efficient and accurate target detection algorithm based on YOLOv8n, termed YOLOv8n-PCPG. (2) Methods: First, a Pinwheel-shaped Convolution (PSConv) is integrated into the backbone to capture directional hyperbolic boundaries. Second, a lightweight CSP-PMSFA module replaces the standard C2f structures in the neck, reducing computational redundancy while preserving feature integrity. Finally, a structurally reparameterized GC-downsampling module is introduced to enhance cross-channel feature interaction without adding inference latency. (3) Extensive experiments on a self-constructed GPR B-scan dataset show that the proposed model achieves a Precision of 97.2%, a Recall of 97.3%, an mAP@0.5 of 98.8%, and an mAP@0.75 of 84.3%. Compared with the baseline YOLOv8n, the parameter count and GFLOPs are reduced by 13.0% and 7.4%, respectively, while achieving a real-time detection speed of 155.78 FPS, outperforming existing mainstream detectors. (4) Conclusions: These results demonstrate that YOLOv8n-PCPG achieves a strong trade-off between detection accuracy and computational efficiency, providing effective technical support for subsurface hyperbolic signature identification.

## 1. Introduction

Ground-Penetrating Radar (GPR) is a widely recognized non-destructive testing (NDT) technology. By emitting high-frequency electromagnetic pulses into subsurface media and receiving reflected echoes from dielectric interfaces, GPR generates radar profiles that characterize subsurface structures, thereby achieving effective imaging and detection of shallow media, buried targets, and structural anomalies [1,2]. Owing to its superior detection efficiency, precise localization capability, strong environmental adaptability, and non-destructive nature, GPR has been extensively deployed in various applications, including military unexploded ordnance (UXO) detection, underground pipeline mapping, pavement distress identification, and subsurface cavity inspection [3,4].

However, traditional GPR image interpretation relies heavily on manual expertise, leading to low processing efficiency and high subjectivity. Furthermore, GPR signals are highly susceptible to harsh environmental interference, such as direct antenna coupling, ground surface reflections, multipath propagation, and clutter scattering. These factors severely exacerbate background noise, attenuate target echoes, and occasionally cause partial signal loss, thereby posing significant challenges for target detection and localization. Early studies primarily adopted the Hough transform [5], least-squares fitting [6], and conventional machine learning techniques [7,8,9,10,11] for hyperbola detection and target recognition. Although these methods have advanced automated processing to some extent, they still suffer from a high reliance on hand-crafted priors, heavy computational overhead, and insufficient robustness in complex field scenarios, making it difficult to achieve an optimal balance between accuracy and efficiency.

In recent years, deep learning has injected new vitality into automated GPR image interpretation. Reviews and geometric studies have highlighted that complex echo morphologies, severe background clutter, and subtle reflection signatures remain critical bottlenecks in GPR data processing [12,13]. Early deep learning applications adapted generic CNNs, Generative Adversarial Networks (GANs), and multi-stage architectures to inspect asphalt pavement moisture damage, structural lining defects, and subsurface anomalies [14,15,16]. Although these pioneering studies demonstrated superior feature representation over hand-crafted priors, their practical engineering deployment was frequently constrained by heavy parameter counts and high computational latency.

To overcome the efficiency bottleneck, single-stage YOLO detectors have gained extensive traction in automated GPR interpretation and engineering inspection [17,18,19,20,21,22,23]. Early implementations applied YOLOv3 for real-time GPR pattern recognition [17], while S. Li et al. demonstrated noticeable performance leaps from YOLOv3 to YOLOv4 and YOLOv5 for GPR concealed crack detection [23]. Subsequent iterations (YOLOv5, YOLOv7, YOLOv8) were progressively extended to detect small targets under low visibility, subway lining defects, underground pipelines, and GPR data classification [18,19,20,21,22,24]. Concurrently, to address extreme target scale variations and feature attenuation, recent research has explored architectural updates—such as network pruning [25] as well as dual-attention BiFPN structures [26] and super-resolution branches (e.g., SuperYOLO [27]) adapted from remote sensing small-target detection—to balance feature representation with computational efficiency.

Despite this progress, a critical synthesis of existing detection methods reveals key limitations: standard convolutional backbones tend to over-smooth fine-grained hyperbolic boundaries under severe clutter, while aggressive network pruning [25] or complex multi-scale feature pyramids [26] frequently induce semantic degradation or computational redundancy in lightweight Neck architectures, leading to high missed detection rates for small-scale hyperbolic signatures.

To address these limitations, this paper adopts the lightweight YOLOv8n as the baseline model and proposes an enhanced architecture, termed YOLOv8n-PCPG, specifically tailored for hyperbolic signature detection in GPR B-scan images. First, the PSConv module is incorporated into the backbone to strengthen feature extraction for slender hyperbolic boundaries and subtle weak-echo details without incurring high computational cost. Second, a newly designed CSP-PMSFA structure replaces the original C2f modules in the Neck network, effectively pruning computational redundancy while preserving both fine-grained spatial details and high-level semantics. Third, a GC-downsampling module replaces standard convolutional downsampling layers in the Neck, facilitating cross-channel feature interaction while maintaining the network’s lightweight nature. Through these synergistic innovations, the proposed model achieves robust hyperbolic signature detection against complex background clutter while strictly balancing detection accuracy and real-time computational efficiency.

## 2. GPR Data Preprocessing

**Figure 1:**
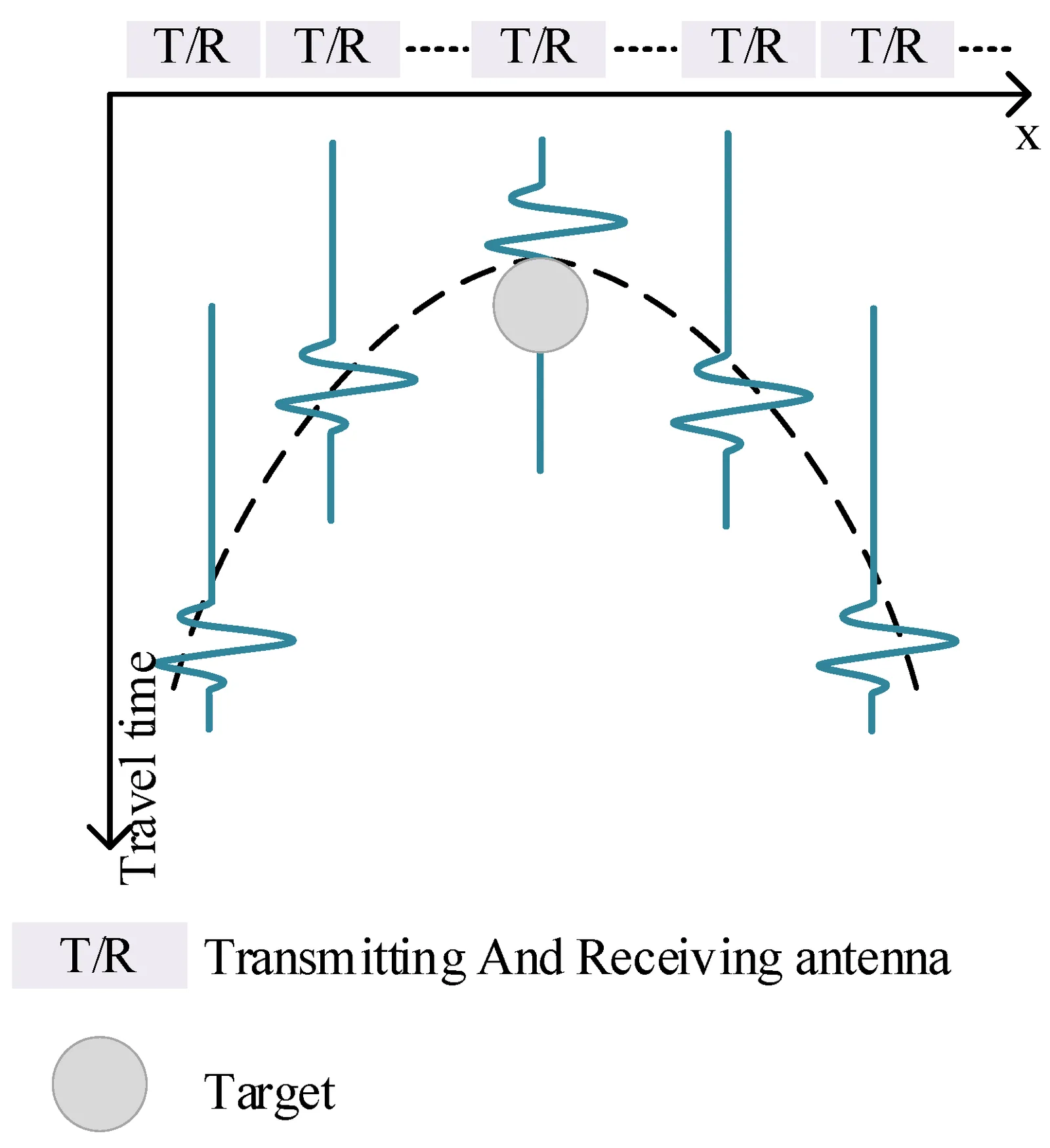
Schematic illustration of hyperbolic signatures in a B-scan image.

In a homogeneous medium, point-like targets or scatterers generate characteristic hyperbolic signatures in Ground-Penetrating Radar (GPR) B-scan images (Figure 1). Alongside target echoes, the received GPR signals inherently capture direct waves induced by antenna coupling and surface reflections. In shallow subsurface surveys with relatively flat terrain, both antenna elevation and ground-coupling conditions remain virtually constant along the survey line. Consequently, direct waves manifest as nearly stationary horizontal background bands in the B-scan profile, whereas target echoes show prominent spatial variations relative to antenna displacement.

Based on these distinct characteristics, an average-trace subtraction method was employed to remove direct waves, thereby suppressing background interference, enhancing target responses, and improving the contrast and interpretability of the B-scan profiles.

Let $s_{i}(t)$ denote the signal of the i-th A-scan trace, where t represents the two-way travel time and N represents the total number of A-scans in the B-scan image. The average reference waveform $s_{i}(t)$ is formulated as:

(1) $$\bar{s}(t)=\frac{1}{N}\sum_{i=1}^{N}s_{i}(t),$$

Accordingly, the preprocessed signal for the i-th trace, denoted as $s{’}_{i}(t)$, is obtained by:

(2) $$s{’}_{i}(t)=s_{i}(t)-\bar{s}(t)=s_{i}(t)-\frac{1}{N}\sum_{k=1}^{N}s_{k}(t)$$

**Figure 2:**
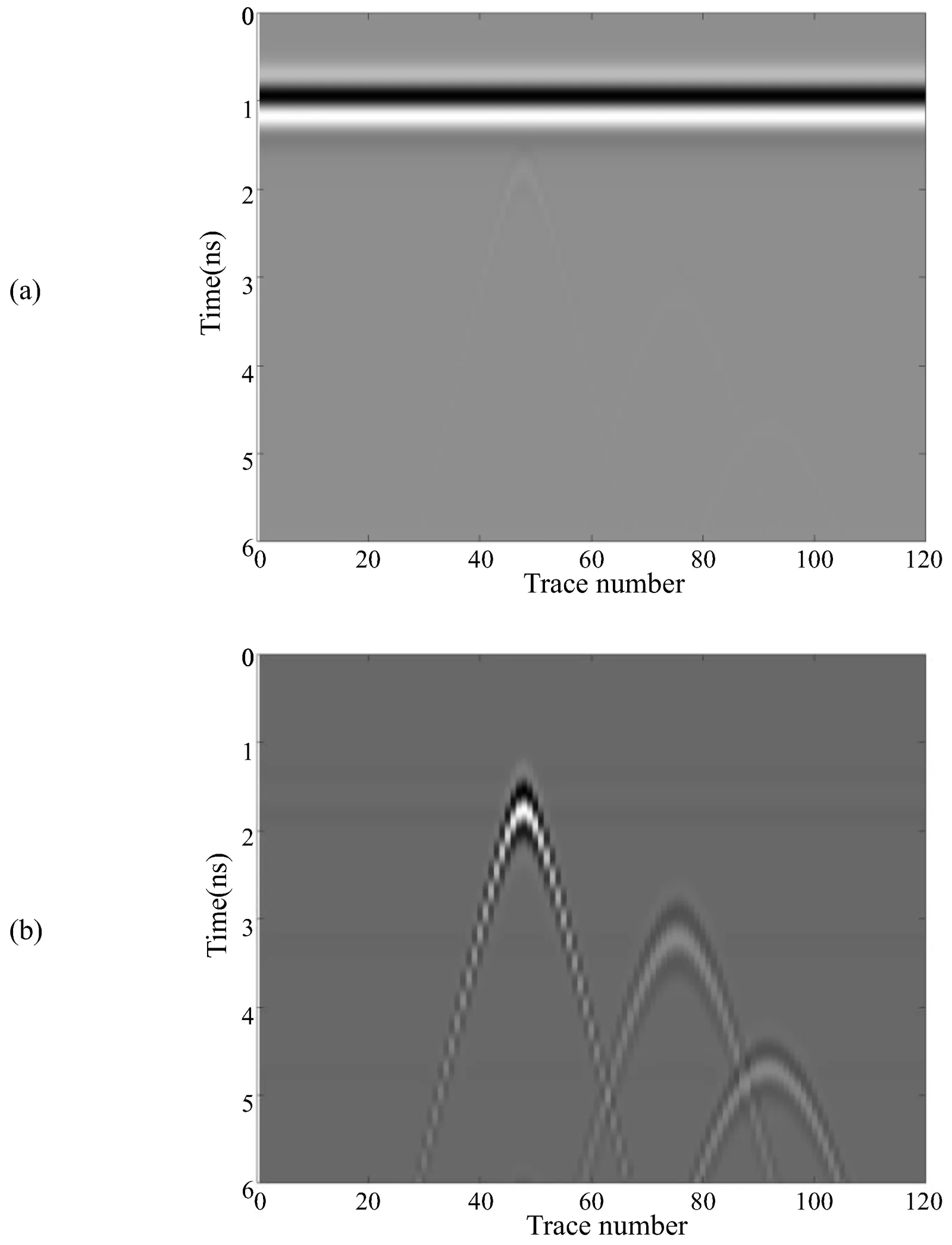
Direct-wave removal result using the average-trace subtraction method: (**a**) Raw GPR B-scan echo data; (**b**) B-scan profile after direct-wave removal.

Figure 2 illustrates the result after direct-wave removal. As can be observed, the background banding associated with direct waves is substantially attenuated, leaving the target hyperbolic signatures more intact and clearly discernible.

## 3. Methods

### 3.1. YOLOv8n-PCPG Models Network Architecture

YOLOv8 is an efficient single-stage object detection algorithm that strikes a good balance between detection accuracy and inference speed. However, in GPR B-scan images, reflection echoes from point-like targets typically manifest as elongated, curved hyperbolic signatures with blurred boundaries. Furthermore, their geometric morphologies and texture distributions vary significantly depending on burial depth, dielectric properties, and background clutter levels.

**Figure 3:**
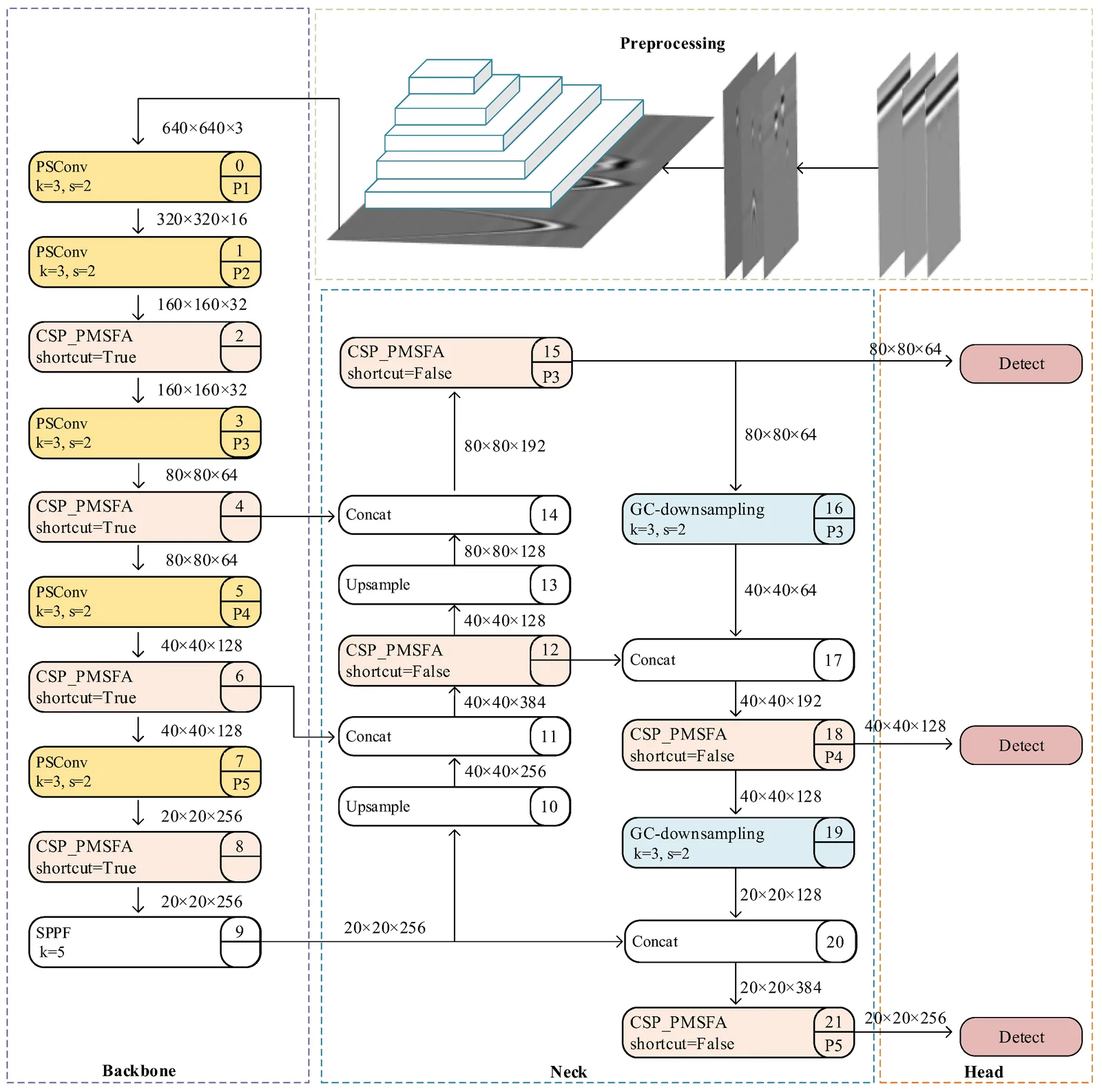
Overall Architecture of the YOLOv8n-PCPG Model.

To enhance the detection performance for hyperbolic targets, this paper adopts YOLOv8n as the baseline model and incorporates the PSConv, CSP-PMSFA, and GC-downsampling modules to improve backbone feature extraction, multi-scale feature aggregation, and contextual information interaction. Consequently, we propose YOLOv8n-PCPG, a lightweight model specifically designed for hyperbola detection in GPR B-scan images. The overall architecture of the proposed model is illustrated in Figure 3.

### 3.2. PSConv

**Figure 4:**
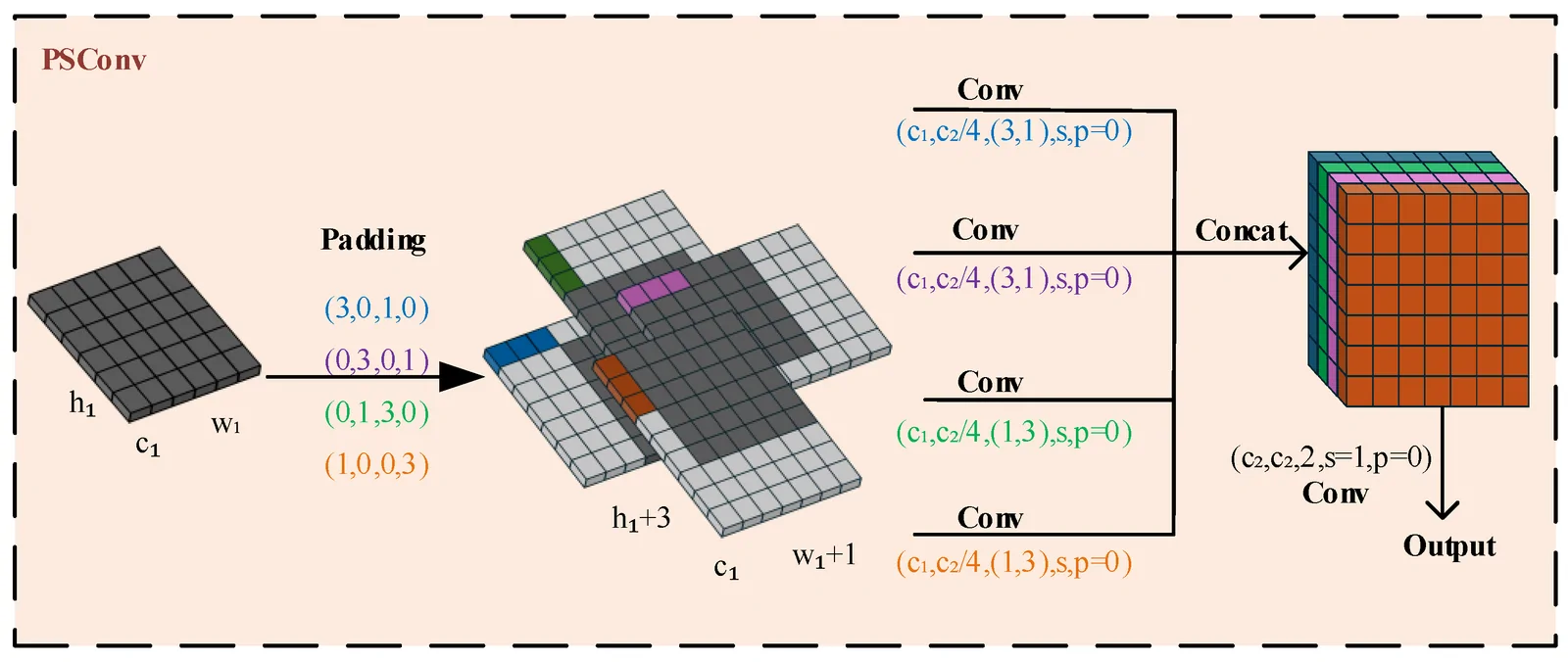
Structure diagram of the proposed PSConv module.

Hyperbolic targets in GPR B-scan images are typically elongated, curved, and highly directional. Conventional convolutions, constrained by fixed receptive-field geometries and limited directional modeling capacity, struggle to adequately capture the critical structural information of these targets. To address this limitation, Pinwheel-shaped Convolution (PSConv) is introduced into the backbone network to replace standard convolutional layers, as illustrated in Figure 4.

Unlike standard convolutions that employ symmetric padding to maintain spatial alignment, PSConv applies direction-specific asymmetric padding combined with 1 × *k* and *k* × 1 convolutional kernels. This design constructs distinct sampling regions along different orientations, thereby enabling more effective feature representation of elongated edges and directional texture patterns. Let $X\in {\mathbb{R}}^{h_{1}\times w_{1}\times c_{1}}$ denote the input feature map, where *h*_1_, *w*_1_, and *c*_1_ Let the weights and biases be represent its height, width, and number of channels, respectively. To enhance training stability and convergence efficiency, each convolution is followed by Batch Normalization (BN) and a SiLU activation function.

The first stage of PSConv consists of four parallel branches. In each branch, an asymmetric padding operation $P\left(left,right,top,bottom\right)$ is first applied to the input, where left, right, top, and bottom denote the number of padded pixels along the respective directions. Subsequently, a 1×k or k×1 convolution is performed on the padded feature map, followed by Batch Normalization (BN) and a SiLU activation function. Accordingly, the parallel convolution operations in the first stage of PSConv can be formulated as follows in Equation (3):

(3) $$\begin{array}{l} X_{1}^{\left(h^{\prime },w^{\prime },c^{\prime }\right)}=\mathrm{SiLU}\left(\mathrm{BN}\left(X_{P(3,0,1,0)}^{\left(h_{1},w_{1},c_{1}\right)}*W_{1}^{\left(3,1,c^{\prime }\right)}\right)\right)\\ X_{2}^{\left(h^{\prime },w^{\prime },c^{\prime }\right)}=\mathrm{SiLU}\left(\mathrm{BN}\left(X_{P(0,3,0,1)}^{\left(h_{1},w_{1},c_{1}\right)}*W_{1}^{\left(3,1,c^{\prime }\right)}\right)\right)\\ X_{3}^{\left(h^{\prime },w^{\prime },c^{\prime }\right)}=\mathrm{SiLU}\left(\mathrm{BN}\left(X_{P(0,1,3,0)}^{\left(h_{1},w_{1},c_{1}\right)}*W_{1}^{\left(1,3,c^{\prime }\right)}\right)\right)\\ X_{4}^{\left(h^{\prime },w^{\prime },c^{\prime }\right)}=\mathrm{SiLU}\left(\mathrm{BN}\left(X_{P(1,0,0,3)}^{\left(h_{1},w_{1},c_{1}\right)}*W_{1}^{\left(1,3,c^{\prime }\right)}\right)\right) \end{array},$$

where $W_{1}^{\left(3,1,c^{\prime }\right)}$ denotes a 3×1 convolutional kernel with c’ output channels, where $c’=c_{1}/4$.

Subsequently, the outputs of the four branches are concatenated along the channel dimension to yield the intermediate feature map as shown in Equation (4):

(4) $${X^{\prime }}^{\left(h^{\prime },w^{\prime },c_{2}\right)}=\mathrm{Cat}\left(X_{1}^{\left(h^{\prime },w^{\prime },c^{\prime }\right)},X_{2}^{\left(h^{\prime },w^{\prime },c^{\prime }\right)},X_{3}^{\left(h^{\prime },w^{\prime },c^{\prime }\right)},X_{4}^{\left(h^{\prime },w^{\prime },c^{\prime }\right)}\right),$$

Finally, to ensure interchangeability between PSConv and standard convolutional layers, an unpadded 2×2 convolutional kernel $W^{\left(2,2,c_{2}\right)}$ is introduced after concatenation for feature fusion and normalization, yielding the final output $Y^{\left(h_{2},w_{2},c_{2}\right)}$ as follows in Equation (5):

(5) $$Y^{\left(h_{2},w_{2},c_{2}\right)}=\mathrm{SiLU}\left(\mathrm{BN}\left({X^{\prime }}^{\left(h^{\prime },w^{\prime },c_{2}\right)}*W^{\left(2,2,c_{2}\right)}\right)\right).$$

### 3.3. CSP-PMSFA

**Figure 5:**
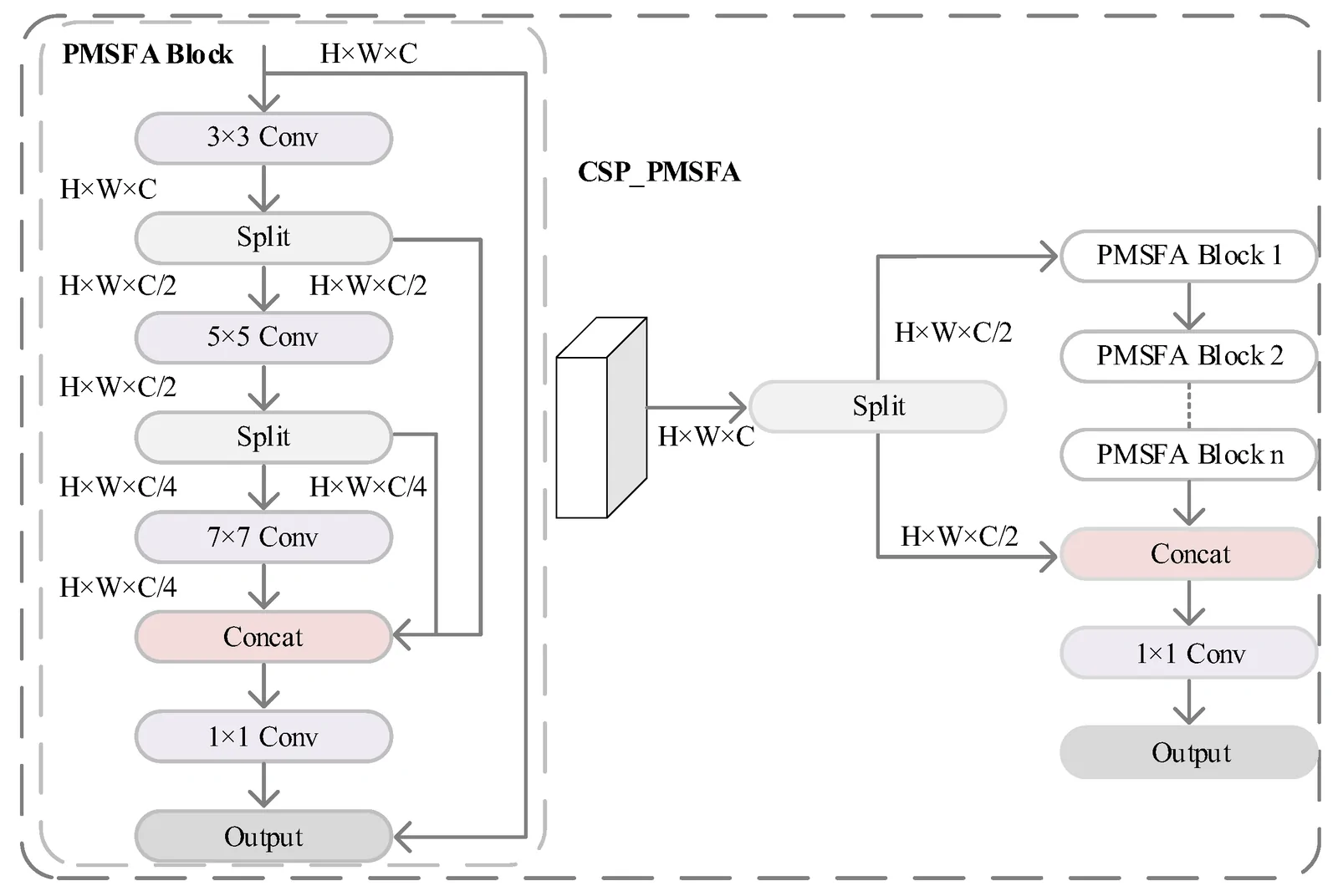
Structure diagram of the proposed CSP-PMSFA module.

Feature maps generated by conventional convolutional networks inherently suffer from information redundancy. Meanwhile, hyperbolic targets in GPR B-scan profiles exhibit significant scale variations and weak local textures. Although expanding the receptive field via large kernels or multi-branch structures enhances multi-scale feature extraction, it inevitably introduces heavy computational overhead, posing significant challenges for lightweight deployment. To strike an optimal balance between feature representation and computational efficiency, we introduce the Cross-Stage Partial Multi-Scale Feature Aggregation (CSP-PMSFA) module. Driven by efficient PartialConv operations, this module processes only a subset of input channels to extract rich multi-scale features while preserving the remaining original context, thereby expanding model capacity at minimal computational cost. The detailed architecture is illustrated in Figure 5.

PMSFA adopts a cascaded progressive architecture. First, a 3×3 convolution extracts initial local features, which are then split into two branches: one is directly preserved, while the other is passed to a 5×5 convolution to expand the receptive field. The feature map from the 5×5 path undergoes a secondary split, where one portion is retained and the remaining portion is processed by a 7×7 convolution to capture broader contextual information. Finally, the preserved features from all intermediate stages are concatenated with the output of the final large-receptive-field branch. A 1×1 convolution subsequently merges cross-channel information, followed by a residual shortcut to the original input to ensure sufficient information flow and stabilize training.

On this basis, CSP-PMSFA incorporates a Cross-Stage Partial (CSP) splitting mechanism to divide input features into two paths: one branch directly retains and propagates original information, while the other passes through PMSFA for multi-scale feature extraction. The two feature streams are subsequently fused to form the final output, effectively reducing computational redundancy while bolstering feature representation capability. This process yields richer and more discriminative features for the subsequent localization and classification of hyperbolic targets.

### 3.4. GC-Downsampling

Target echoes in B-scan images are highly susceptible to clutter and noise interference. However, the local receptive fields in standard YOLOv8 constrain cross-scale feature interactions. To address this, we introduce a GC-downsampling module to align feature-map resolutions, enhance interference immunity and strengthen the robustness of radar hyperbola detection.

**Figure 6:**
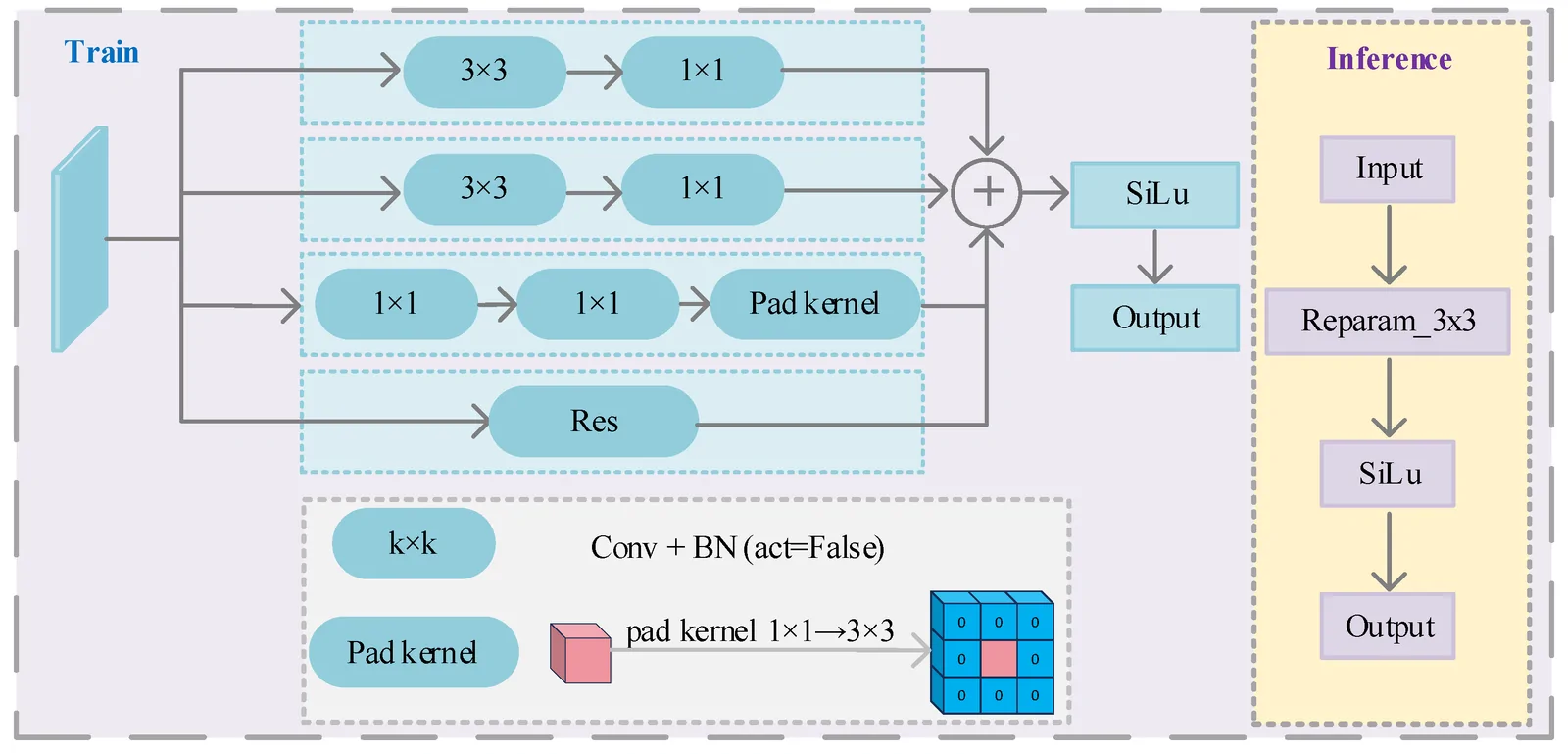
Structure diagram of the proposed GC-downsampling module.

The structural reparameterization mechanism of the proposed module operates as follows: During training, a multi-branch convolutional architecture captures richer feature representations via parallel branches, where Batch Normalization (BN) is applied after each convolution to normalize channel distributions and perform learnable affine transformations, thereby stabilizing training and accelerating convergence. During inference, structural reparameterization consolidates these branches—along with the BN parameters—into a single 3×3 convolutional layer, significantly reducing computational overhead and latency while preserving accuracy. The module architecture is illustrated in Figure 6.

For a sequential path consisting of a 3×3 convolution and a 1×1 convolution without intermediate batch normalization or activation layers, the two layers can be merged into a single 3×3 convolution. Let the weights and biases be $W_{3\times 3}\in {\mathbb{R}}^{C_{out1}\times \left(C_{in}\times 3\times 3\right)}$, $B_{3\times 3}\in {\mathbb{R}}^{C_{out1}}$, $W_{1\times 1}\in {\mathbb{R}}^{C_{out2}\times \left(C_{out1}\times 3\times 3\right)}$, and $B_{1\times 1}\in {\mathbb{R}}^{C_{out2}}$. For an input x, the output y is formulated using the im2col operator $I\left(\cdot \right)$ and matrix multiplication:

(6) $$\begin{array}{cc} y & =W_{1\times 1}*(W_{3\times 3}*x+B_{3\times 3})+B_{1\times 1}\\ & =W_{1\times 1}\cdot I_{2}(W_{3\times 3}\cdot I_{1}(x)+B_{3\times 3})+B_{1\times 1}\\ & =W_{1\times 1}\cdot I_{2}(W_{3\times 3}\cdot I_{1}(x))+W_{1\times 1}\cdot B_{3\times 3}+B_{1\times 1} \end{array},$$

where $I\left(\cdot \right)$ transforms the input tensor into a two-dimensional matrix according to the shape of the convolution weights.

Since $W_{3\times 3}\cdot I_{1}\left(x\right)$ has a shape of $C_{out1}\times \left(H^{\prime }\times W^{\prime }\right)$ and $W_{1\times 1}$ has a shape of $C_{out2}\times \left(C_{out1}\times 1\times 1\right)$, we have $W_{1\times 1}\cdot I_{2}\left(W_{3\times 3}\cdot I_{1}\left(x\right)\right)=W_{1\times 1}\cdot W_{3\times 3}\cdot I_{1}\left(x\right)$. Substituting this into Equation (7) yields:

(7) $$\begin{array}{cc} y & =W_{1\times 1}\cdot I_{2}(W_{3\times 3}\cdot I_{1}(x))+W_{1\times 1}\cdot B_{3\times 3}+B_{1\times 1}\\ & ={W^{\prime }}_{3\times 3}\cdot I_{1}(x)+{B^{\prime }}_{3\times 3,} \end{array}$$

where the merged weight ${W^{\prime }}_{3\times 3}$ and bias ${B^{\prime }}_{3\times 3}$ represent the newly formed convolutional weight and integrated bias.

Thus, the sequential connection of a 3×3 convolution and a 1×1 convolution can be seamlessly collapsed into a single equivalent 3×3 convolution without performance loss.

Likewise, sequential 1×1 convolutions and identity mapping shortcuts can all be transformed into an equivalent 3×3 kernel by zero-padding the spatial surroundings or setting diagonal center weights to 1.

Upon reparameterizing each path into a 3×3 convolutional layer, we obtain n weights $W_{i}$ and n biases $B_{i}$. For an input x, the fused output of the parallel multi-path structure is expressed as Equation (8):

(8) $$\begin{array}{cc} y & =\sum_{i=1}^{n}\left(W_{i}*x+B_{i}\right)\\ & =\left(\sum_{i=1}^{n}W_{i}\right)*x+\sum_{i=1}^{n}B_{i},\\ & =W^{\prime }*x+B^{\prime } \end{array}$$

where $W^{\prime }$ and $B^{\prime }$ denote the equivalent convolutional weights and bias after fusion, respectively.

Thus, the multi-branch architecture during training can be losslessly converted into a single 3×3 convolutional layer for inference, significantly reducing computational overhead while preserving feature representation capability.

## 4. Experiments

### 4.1. Dataset Description

This section describes the synthetic GPR dataset constructed for model training and evaluation. To ensure reproducibility, all numerical and electromagnetic parameters used in the gprMax 3.0 modeling are explicitly detailed in Table 1 and Table 2. Specifically, Table 1 outlines the numerical simulation domain, grid discretization, boundary conditions, and GPR antenna acquisition configurations.

To evaluate target signature recognition under controlled baseline conditions, a synthetic dataset comprising 3000 GPR B-scan profiles was generated using gprMax 3.0 over a macroscopically uniform soil background without environmental clutter or additive noise. As summarized in Table 2, the dataset incorporates four distinct target categories: air-filled PVC tubes (PVC-air), water-filled PVC tubes (PVC-water), Perfect Electric Conductors (Pec, representing steel rebar), and cavities (Void).

Target parameter diversity is achieved by independently sampling physical dimensions, burial depths, and horizontal spatial locations from continuous uniform distributions within the specified parameter bounds, encompassing single-target, two-target, and three-target configurations. All targets are oriented with their longitudinal axes parallel to the *z*-axis (orthogonal to the B-scan survey trajectory along the *x*-axis). Geometric collision detection is enforced during generation to ensure physical non-overlap while capturing multi-target wave interference and hyperbolic superposition. Waveform response characteristics across different materials are detailed in Table 3, and the complete dataset generation Python script using the gprMax API is provided as Supplementary Material (see Supplementary Material File S1). It should be noted that the present dataset is entirely constructed based on numerical simulations and does not fully encompass real-world environmental complexity.

To establish a standardized ground truth, bounding boxes were manually labeled via LabelImg(v1.8.1) for each target. Each bounding box is strictly defined to enclose the primary hyperbolic reflection signature. For multi-target scenarios featuring wavefield interference, intersecting regions are retained within both corresponding bounding boxes without mutual suppression, enabling the network to learn feature representations of overlapping wave patterns.

To enhance data diversity, single-target, multi-target, and multi-material burial forms were further configured in the setting of buried targets, as shown in Table 4. To evaluate the model performance without data leakage, dataset partitioning was executed strictly at the independent simulation scenario level. The 3000 GPR B-scan profiles correspond to 3000 distinct numerical simulations featuring independently randomized target combinations, scales, and spatial locations. These independent scenarios were randomly divided into training, validation, and test sets in a 7:2:1 ratio (2100, 600, and 300 unique B-scans, respectively). Since each B-scan profile may contain 1 to 3 targets, Table 5 details the distribution of target instances across the partitioned subsets.

### 4.2. Environment and Evaluation Metrics

All experiments were conducted on a single NVIDIA GeForce RTX 3090 GPU equipped with PyTorch 2.2.2, CUDA 11.8, and Python 3.8.2. Model performance was evaluated using Precision (P), Recall (R), mean Average Precision at an IoU threshold of 0.5 (mAP@0.5), mean Average Precision at an IoU threshold of 0.75 (mAP@0.75), mean Average Precision across IoU thresholds from 0.5 to 0.95 (mAP@0.5:0.95), Frames Per Second (FPS), number of parameters (Params), and floating-point operations (GFLOPs). Note that the evaluation metrics in this study (mAP, Precision, Recall) quantify the model’s accuracy in hyperbola detection within B-scan images, while the reported real-time inference speed (FPS) is evaluated on an NVIDIA GeForce RTX 3090 GPU, representing performance under high-performance computing setups rather than CPU devices. The detailed calculation formulas are defined in Equations (9)–(12):

(9) $$P=\frac{TP}{TP+FP},$$

(10) $$R=\frac{TP}{TP+FN},$$

(11) $$AP=\int_{0}^{1}P(R)dR,$$

(12) $$mAP=\frac{\sum_{i=1}^{K}AP_{i}}{K}.$$

where TP, FP, and FN denote true positives, false positives, and false negatives, respectively; K represents the total number of classes.

### 4.3. Implementation Details and Baseline Configurations

To ensure a fair and reproducible evaluation, YOLOv8n was selected as the primary baseline model for structural improvements. All evaluated architectures were categorized into two main groups based on their training paradigms.

For the YOLO family (YOLOv5n, YOLOv8n, YOLOv10n, YOLOv11n, and YOLOv12n), models were implemented using their official open-source repositories—including the Ultralytics Framework (for YOLOv5, YOLOv8, and YOLOv11), the official YOLOv10 Repository, and the official YOLOv12 Repository. All YOLO variants were trained from scratch (random weight initialization without pre-trained weights) for 100 epochs under identical hyperparameter settings.

For non-YOLO baseline detectors (RTMDet-tiny, Faster R-CNN, and TOOD), experiments were integrated and executed using the OpenMMLab MMDetection (v3.0) framework. To respect their distinct architectural characteristics, each non-YOLO model strictly followed its official default configuration and ImageNet pre-trained initialization scheme. A complete summary of the baseline configurations and hyperparameter settings across all evaluated models is presented in Table 6.

### 4.4. Ablation Study

To quantitatively evaluate the impact of each proposed module on hyperbola detection performance in B-scan images, ablation experiments were conducted based on the YOLOv8n baseline using a controlled variable approach. Experiments A–C verify the effectiveness of individual modules, Experiments D–F analyze the synergistic effects of pairwise combinations, and Experiment G assesses the overall performance improvement. All quantitative metrics are reported as Mean ± Standard Deviation across multiple independent runs (seeds 0, 42, and 1024). The results are summarized in Table 7.

Experiment A shows that adding PSConv improves Precision, Recall, mAP@0.5, and mAP@0.5:0.95 by 1.0, 0.5, 0.8, and 1.4 percentage points, respectively; while slightly reducing parameters to 2.96 M, it incurs only a minor increase of 0.3 GFLOPs. Experiment B demonstrates that CSP-PMSFA improves P and R by 0.8 and 0.7 percentage points, while boosting mAP@0.5 and mAP@0.5:0.95 by 0.8 and 1.4 percentage points; notably, it reduces parameters and GFLOPs by approximately 13.6% and 12.3%, highlighting a favorable trade-off between accuracy and efficiency. Experiment C indicates that without introducing additional parameters or GFLOPs, the GC-downsampling module improves mAP@0.5 and mAP@0.5:0.95 by 0.5 and 1.2 percentage points, respectively, while boosting FPS to 154.96, validating its efficacy in enhancing feature representation and computational speed.

Multi-module combination experiments further confirm the strong complementarity among these modules. Ultimately, the final model (Experiment G), which integrates PSConv, CSP-PMSFA, and GC-downsampling, achieves superior performance—improving P, R, mAP@0.5, mAP@0.75, and mAP@0.5:0.95 by 3.6, 3.2, 2.4, 12.6, and 7.1 percentage points over the baseline, respectively. Meanwhile, it reduces parameters and GFLOPs by 13.0% and 7.4% while maintaining a high inference speed of 155.78 FPS, thus achieving an optimal balance between accuracy and computational cost.

In summary, the multi-seed ablation results strongly validate the effectiveness and synergistic advantages of the proposed modules, confirming that the final model delivers statistically robust performance gains for B-scan hyperbola detection. Notably, while the model achieves high accuracy at standard thresholds (mAP@0.5), performance declines under stricter IoU criteria (mAP@0.75 and mAP@0.5:0.95). This gap highlights the inherent challenge of precise bounding-box alignment for diffuse hyperbolic signatures under strict IoU constraints, rather than a mere metric artifact, indicating a key direction for future architecture refinement.

### 4.5. Comparative Experiments

#### 4.5.1. Ablation Study on Average-Trace Subtraction Preprocessing

To quantitatively evaluate the necessity and independent contribution of average-trace subtraction in our GPR detection pipeline, an ablation experiment was conducted comparing raw profiles with those after average-trace subtraction. To eliminate potential annotation biases caused by target ambiguity in raw data, we mapped the high-fidelity ground-truth bounding boxes generated from the filtered images directly back to the raw profiles in a 1-to-1 correspondence.

As shown in Table 8, YOLOv8-PCPG was evaluated under identical hyperparameter settings on both datasets. The experimental results demonstrate a substantial performance drop across all metrics when operating on raw data, primarily due to the strong direct-wave masking effect and horizontal clutter. The application of average-trace subtraction significantly enhances target boundary visibility, enabling the detection backbone to achieve superior feature extraction.

#### 4.5.2. Comparative Analysis of Feature Extraction Mechanisms for GPR Hyperbolic Signatures

To explicitly demonstrate the capability of PSConv in capturing GPR hyperbolic signatures, controlled experiments were conducted at the downsampling nodes (layers 0, 1, 3, 5, and 7) of the YOLOv8 backbone. Under identical hyperparameter setups and across three random seeds (Seed = 0, 42, 1024), PSConv was benchmarked against Haar Wavelet Downsampling (HWD), Space-to-Depth Convolution (SPD), and Wavelet Pooling. The quantitative results are summarized in Table 9.

As summarized in Table 9, PSConv achieves the optimal overall performance, remarkably outperforming competing downsampling methods with an mAP@0.75 of 0.7418 and the fastest inference speed of 104.79FPS. This superior accuracy stems from its multi-scale receptive field slicing and parallel convolution design, which effectively preserves both localized apices and faint, continuous hyperbolic tails in GPR B-scans during stride-2 feature reduction. Meanwhile, PSConv maintains a balanced computational footprint (8.4 GFLOPs) and high throughput, confirming its capability to balance fine-grained geometric feature extraction with real-time subsurface inspection efficiency.

#### 4.5.3. Comparative Evaluation of CSP-PMSFA Aggregation Module

To ensure strict control of variables, CSP-PMSFA was benchmarked against representative feature aggregation modules—encompassing lightweight re-parameterization design (RepNCSPELAN-OREPA based on Online Conv Re-parameterization) and directional/multi-scale feature extraction architectures (CSP-EDLAN based on DualConv, and RepNCSPELAN-DRB based on Dilated Reparam Block)—by systematically swapping each into identical backbone (layers 2, 4, 6, 8) and neck (layers 12, 15, 18, 21) feature aggregation nodes across three random seeds (Seed = 0, 42, 1024). The quantitative results are summarized in Table 10 as Mean ± Std.

As detailed in Table 10, CSP-PMSFA achieves superior overall detection performance across all primary metrics, attaining the highest Recall (0.9519), mAP@0.5 (0.9726), mAP@0.75 (0.7401), and mAP@0.5:0.95 (0.6324). This enhancement stems from the synergy between cross-stage feature reuse and multi-scale phase attention, which effectively reinforces weak, continuous hyperbolic diffraction tails while filtering out complex subsurface clutter.

Furthermore, CSP-PMSFA exhibits exceptionally low standard deviations in Precision and Recall, demonstrating outstanding training stability against random initialization and data perturbation. Notably, despite a slight increment in parameters (2.60 M) and computational complexity (7.1 GFLOPs), CSP-PMSFA yields the fastest inference speed (127.68 FPS), demonstrating that its single-path latency-friendly design effectively avoids the runtime multi-branch execution overhead common in heavy re-parameterization or complex multi-kernel structures, thereby achieving the optimal trade-off between detection precision and real-time operational efficiency for GPR detection.

#### 4.5.4. Comparison with Existing Detection Models

To verify the effectiveness of the proposed method for hyperbola detection in B-scan images, comparative experiments were conducted against several mainstream object detection algorithms, including the lightweight YOLOv5n, YOLOv8n, YOLOv10n, YOLOv11n, and YOLOv12n series, as well as classic baseline models such as Faster R-CNN, TOOD, and RTMDet-tiny. To ensure a fair comparison and eliminate random initialization bias, all models were trained and evaluated under identical dataset splits, hyperparameter configurations, and evaluation protocols across three distinct random seeds (Seed = 0, 42, 1024). Performance is reported as Mean ± Standard Deviation across key metrics, including mAP@0.5, mAP@0.75, mAP@0.5:0.95, Parameters, GFLOPs, and FPS. The experimental results are summarized in Table 11.

The results indicate that the proposed method achieves superior detection performance while maintaining a low parameter count and computational complexity, with mAP@0.5 and mAP@0.5:0.95 reaching 0.9888 and 0.6894, respectively. Specifically, compared with Faster R-CNN, the parameter count and GFLOPs are reduced by approximately 93.7% and 95.8%, respectively. Compared with the representative lightweight model YOLOv5n, our method achieves a 5.73 percentage point gain in mAP@0.5:0.95 under comparable parameter and GFLOP constraints. This demonstrates that the proposed method effectively balances shallow detail extraction, multi-scale feature fusion, and global context modeling, thereby exhibiting superior hyperbola detection performance and lightweight efficacy specifically under synthetic GPR profile environments.

### 4.6. Generalization Experiment

To evaluate model robustness and cross-domain applicability, we conducted generalization tests on an independent GPR dataset (A Ground Penetrating Radar dataset for Deep Learning applications). The dataset comprises 554 B-scan images of underground cavities (‘Cavity’) collected across infrastructure projects in Morocco (2019–2024). Experiments were executed across three random seeds (seed = 0, 42, 1024), with results reported as Mean ± Std in Table 12.

As shown in Table 12, YOLOv8n-PCPG outperforms the baseline across all metrics. Precision and Recall increase by 6.41% and 4.95%, while mAP@0.75 and mAP@0.5:0.95 improve by 4.30% and 5.14%, respectively, indicating a certain degree of stability and generalization capability in real-world GPR applications.

Nevertheless, a practical limitation lies in the constrained scale and target diversity of current field datasets. While the evaluated dataset focuses primarily on underground cavities, real-world GPR inspection encounters highly heterogeneous soil environments (e.g., varying clay contents and moisture levels) and diverse subsurface targets such as pipelines, rebar, and geological voids. Therefore, further validation across broader soil types and multi-class target scenarios is required to fully confirm the model’s operational adaptability.

### 4.7. Visualization Analysis

To gain a more intuitive assessment of the proposed model’s practical performance in B-scan hyperbola detection, visualization analysis was conducted on the test set, with the quantitative results summarized in Table 13. The findings indicate that YOLOv8n-PCPG consistently outperforms the baseline model in both class-specific AP_0.5_ and overall mAP@0.5, demonstrating superior feature recognition capability within the simulated evaluation scenarios.

**Figure 7:**
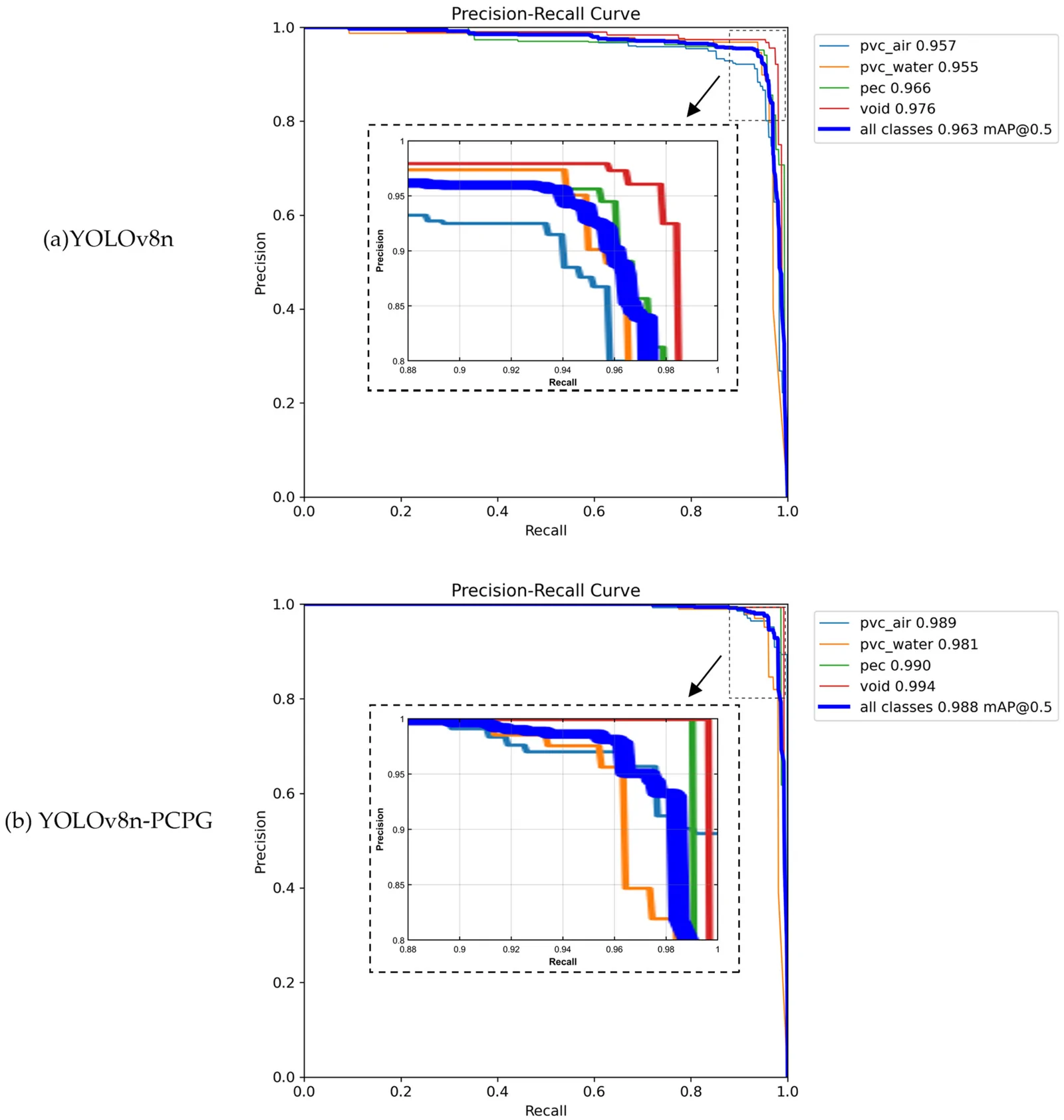
P-R curve comparison between YOLOv8n and YOLOv8n-PCPG: (**a**) P-R curve of YOLOv8n; (**b**) P-R curve of YOLOv8n-PCPG.

Furthermore, as depicted in the Precision–Recall (P-R) curves in Figure 7, the proposed model achieves a better overall trade-off between precision and recall than standard YOLOv8n, where (a) and (b) present the P-R curves of YOLOv8n and YOLOv8n-PCPG, respectively.

**Figure 8:**
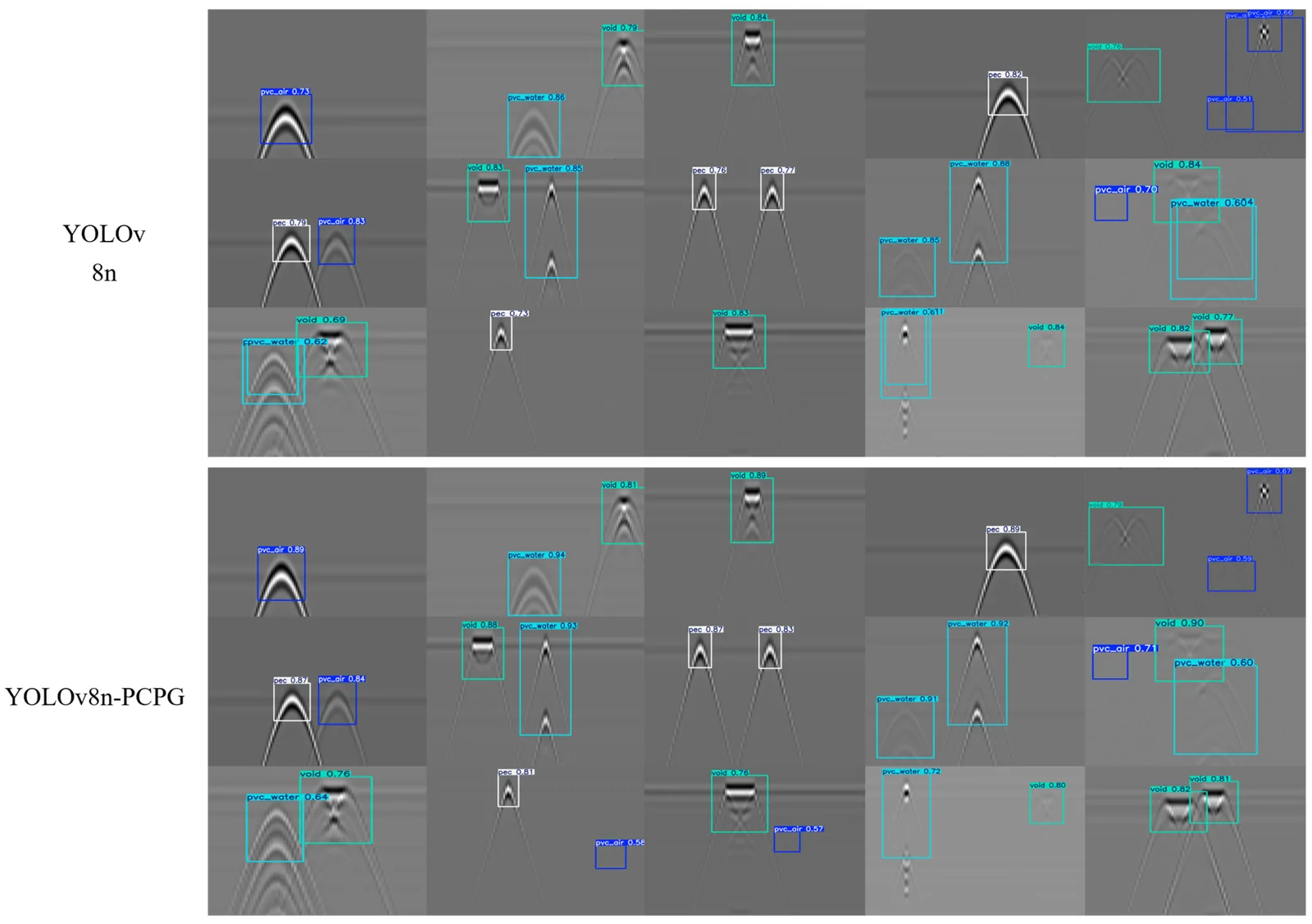
Qualitative comparison of hyperbolic signature detection results on GPR B-scan profiles among different models.

The detection result comparison is illustrated in Figure 8. As observed, compared with the baseline YOLOv8n model, YOLOv8n-PCPG achieves higher detection accuracy for hyperbolic, identifying hyperbolic signatures more accurately while effectively reducing both missed detections and false alarms.

**Figure 9:**
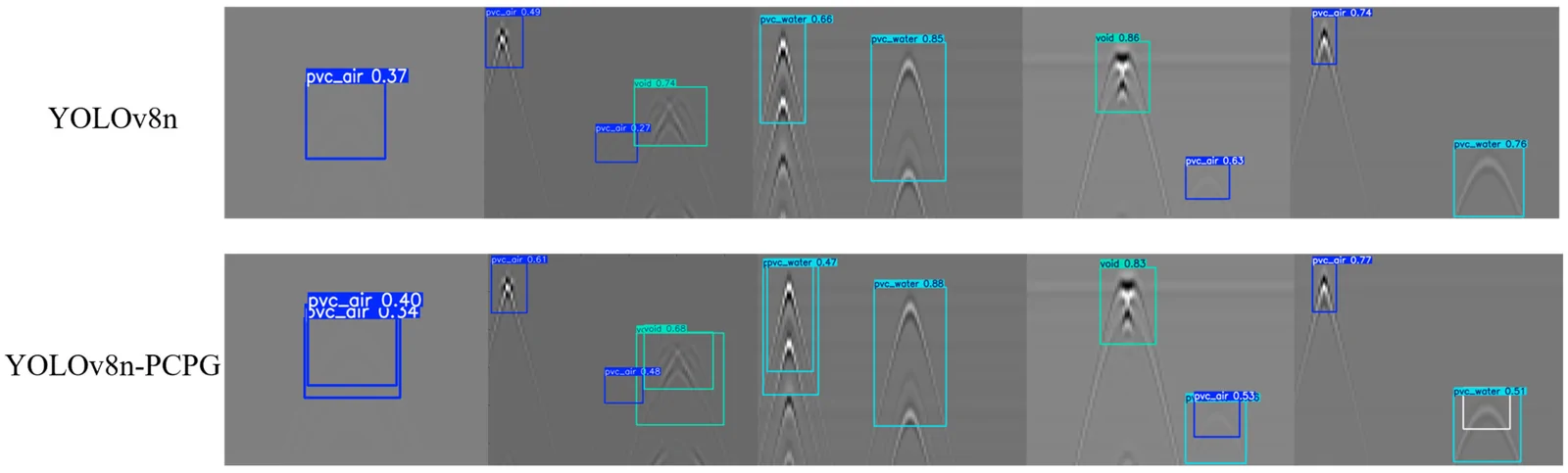
Failure Case Analysis of YOLOv8n-PCPG Model.

Although the proposed YOLOv8n-PCPG significantly suppresses missed detections and false alarms compared with the baseline YOLOv8n, certain detection errors still persist, as illustrated in Figure 9. Specifically, when target echoes are extremely weak and hyperbolic features become indistinct or vague, it becomes challenging for the model to extract sufficiently discriminative features. Under such conditions, the model may occasionally misinterpret faint background artifacts or subtle signal fluctuations as targets, leading to false positives or redundant bounding box detections.

Furthermore, while the proposed architecture is evaluated on multi-class synthetic GPR B-scan images, its generalization capability has only been benchmarked on a single-class public dataset (‘Cavity’ from infrastructure projects in Morocco). Because comprehensive multi-class public field GPR datasets remain scarce, the model’s cross-domain performance on other real-world targets (e.g., pipelines and rebar) under heterogeneous soil environments remains to be further validated. To address this constraint without relying on scarce field annotations, future work will explore advanced physics-informed data augmentation, domain adaptation, and realistic clutter simulation techniques to broaden the scope of multi-target and complex-environment evaluations.

**Figure 10:**
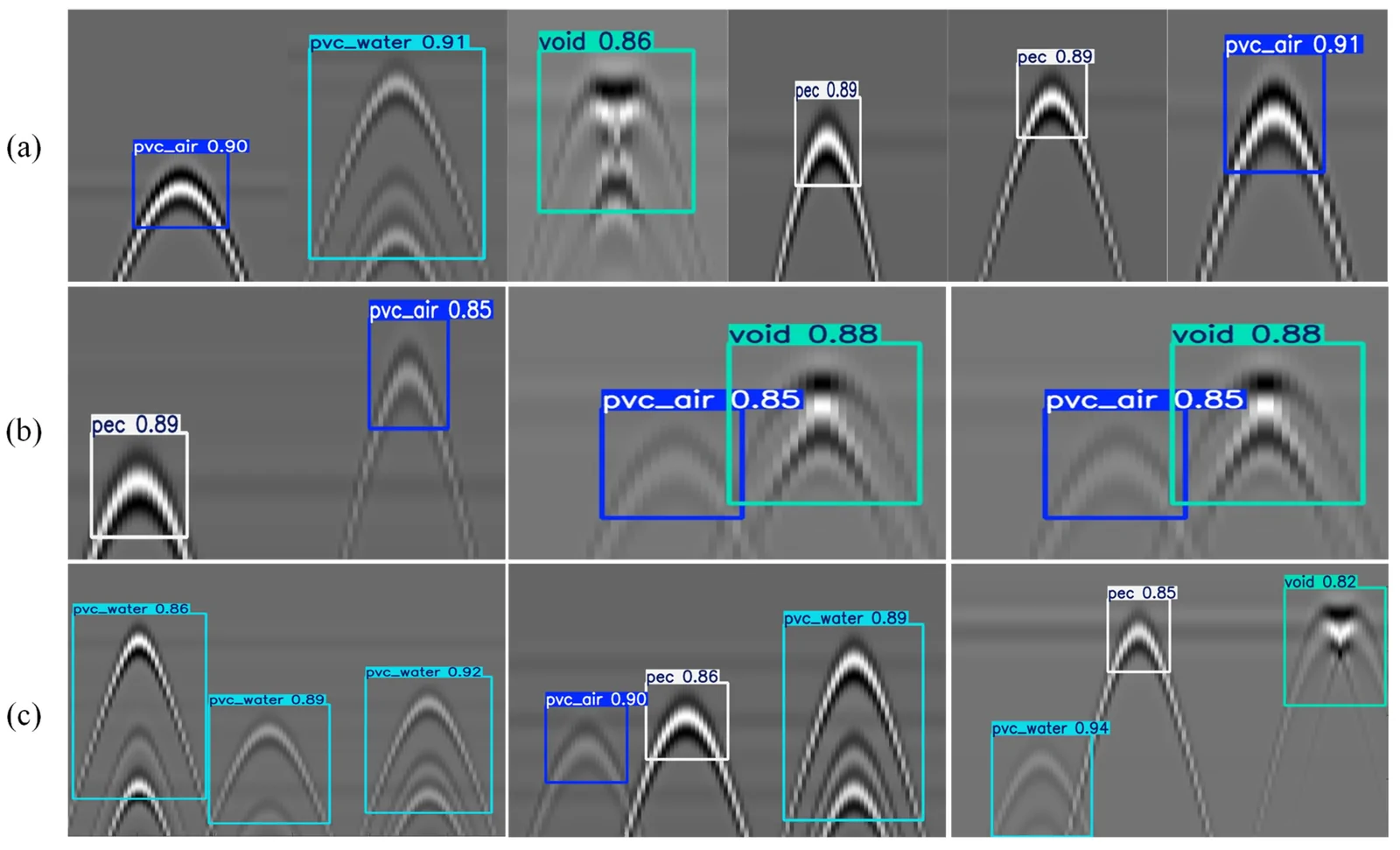
Examples of test detection results: (**a**) Detection example of a single target; (**b**) Detection example of dual targets; (**c**) Detection example of triple targets.

To further demonstrate the model’s robustness under different spatial complexity, Figure 10 illustrates representative detection results under varying target densities, where (a) presents a single target, (b) displays dual targets, and (c) shows triple targets. The results demonstrate that YOLOv8n-PCPG consistently achieves accurate detection of hyperbolic targets in GPR B-scan images across different density scenarios, highlighting the superior stability and adaptability of the proposed model.

## 5. Conclusions

This paper presents YOLOv8n-PCPG, an efficient and lightweight deep learning framework tailored for hyperbolic pattern detection in GPR B-scan profiles. By synergistically integrating PSConv, CSP-PMSFA, and GC-downsampling modules, the proposed model achieves superior detection performance—improving Precision, Recall, mAP@0.5, mAP@0.75, and mAP@0.5:0.95 by 3.6, 3.2, 2.4, 12.6, and 7.1 percentage points over the baseline YOLOv8n, respectively. Crucially, these accuracy enhancements are accomplished while reducing total parameters by 13.0% and GFLOPs by 7.4%, alongside maintaining a high real-time inference speed of 155.78 FPS. Multi-seed ablation results confirm that YOLOv8n-PCPG achieves an optimal trade-off between detection accuracy and computational overhead, offering a highly robust and practical lightweight baseline for automated subsurface hyperbolic signature detection.

However, while the preliminary generalization of YOLOv8n-PCPG has been validated on a real-world GPR Cavity dataset, the current evaluation remains bounded by limited field data scale, uniform soil conditions, and specific target categories. Real-world subsurface conditions—characterized by complex soil heterogeneity, moisture variations, diverse target types (e.g., pipes, rebar), and instrumental noise—present operational challenges that require broader verification. Additionally, the reported real-time inference speed specifically reflects performance under the desktop RTX 3090 benchmarking environment. Future research will focus on expanding model generalization and practical deployment under complex field conditions. On the one hand, domain adaptation techniques (e.g., GANs) and physics-informed data augmentation will be explored to enrich training distributions. On the other hand, incorporating larger-scale field-measured datasets encompassing heterogeneous soil environments and multi-class subsurface targets, alongside lightweight optimization for edge devices, will be essential to advance the practical adaptability of the proposed framework.

## Figures and Tables

**Table 1:** Numerical simulation domain and acquisition configurations in gprMax 3.0.

Parameter	Configuration
Simulation Domain Size (Excl. PML)	(x, y, z): (2.5 m, 1.0 m, 0.001 m)
Spatial Mesh Size	(∆x, ∆y, ∆z): (0.001 m, 0.001 m, 0.001 m)
Boundary Conditions	Perfectly Matched Layer(PML)
Time Window	5.58 ns
Center Frequency	1.5 GHz
Antenna Type	Hertzian dipole (Z-directed)
Excitation Waveform	Ricker Wavelet
Spatial Trace Interval	0.02 m
Tx–Rx Configuration	0.01 m

**Table 2:** Geometric dimensions and electromagnetic properties of buried targets and background media.

Material	Shape	Dimensions (m)	Wall/Height (m)	Relative Permittivity ($\varepsilon_{r}$)	**σ** (S/m)	Spatial/Depth Bounds (m)
Soil Background	Block background	—	Depth: 0.900	4	0.01	x: [0.0, 2.5] y: [0.0, 0.90]
Pvc-air	Concentric Cylinder	Outer Radius: 0.010–0.030	Wall: 0.005	Wall: 3, Internal Air: 2	Wall: 0, Air: 0	x: [0.20, 2.30] y: [0.20, 0.70]
Pvc-water	Concentric Cylinder	Outer Radius: 0.010–0.030	Wall: 0.005	Wall: 3, Internal- Water: 81	Wall: 0, Water: 0.03	x: [0.20, 2.30] y: [0.20, 0.70]
Pec	Cylinder	Radius: 0.010–0.030	—	∞	∞	x: [0.20, 2.30] y: [0.20, 0.70]
Void	Cuboid	Width: 0.100–0.300	Height: 0.050–0.150	1	0	x: [0.20, 2.30] y: [0.20, 0.70]

**Table 3:** Target waveform features in GPR B-scan profiles.

Sample	Category	Features
** 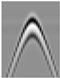 **	Pvc-air	The top of the parabola corresponds to the target position, with clear white-black-white stripes visible and distinct hyperbolic echo characteristics.
** 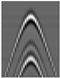 **	Pvc-water	A series of clear and regular parabolic echo characteristics can be observed from top to bottom.
** 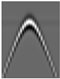 **	Pec	The top of the parabola corresponds to the target position, with clear black-white-black stripes visible.
** 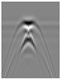 **	Void	A series of parabolic echo characteristics with distribution patterns.

**Table 4:** Simulation setup and environmental configurations.

Target Quantity	Target Type	Background	Target Location Range [Start, End]
Single target	Pvc-air, Pvc-water, Pec, Void	Uniform medium (soil)	Horizontal position: [0.10, 0.30] Depth: [0.05, 0.15]
Mixed dual targets			
Mixed three targets			

**Table 5:** Distribution of target instances in the partitioned datasets.

Target Class	Dataset		
	Training Set	Validation Set	Test Set
Pvc-air	979	281	143
Pvc-water	803	220	109
Pec	962	277	143
Void	889	262	133

**Table 6:** Detailed Training Configurations and Hyperparameter Settings.

Category	Model Architecture	Pre-Trained Weights	Input Resolution	Batch Size	Epochs	Optimizer & Learning Rate
YOLO Series	YOLOv5n/v8n/v10n/v11n/v12n	None (From Scratch)	640 × 640	64	100	SGD(lr = 0.01, mom = 0.937; wd = 0.0005)
Baselines	RTMDet-tiny	ImageNet Pre-trained	640 × 640	16	300	AdamW(lr = 0.004, wd = 0.05)
	Faster R-CNN (ResNet-50)	ImageNet Pre-trained	1333 × 800	8	12	SGD(lr = 0.02, mom = 0.9; wd = 0.0001)
	TOOD (ResNet-50)	ImageNet Pre-trained	1333 × 800	8	12	SGD(lr = 0.01, mom = 0.9; wd = 0.0001)

**Table 7:** Ablation study of the proposed modules on YOLOv8n baseline (reported as Mean ± SD across seeds 0, 42, and 1024).

YOLOv8	PS Conv	CSP-PMSFA	GC-Downsampling	Params (M)	G- FLOPs	P	R	mAP @0.5	mAP @0.75	mAP@ 0.5:0.95	FPS
✓	—	—	—	3.01	8.1	0.9411 ± 0.0069	0.9447 ± 0.0072	0.9647 ± 0.0017	0.7069 ± 0.0240	0.6180 ± 0.0083	125.72 ± 0.0513
A	✓	—	—	2.96	8.4	0.9506 ± 0.0041	0.9500 ± 0.0046	0.9724 ± 0.0015	0.7418 ± 0.0252	0.6321 ± 0.0044	104.79 ± 0.7400
B	—	✓	—	2.60	7.1	0.9488 ± 0.0011	0.9519 ± 0.0013	0.9726 ± 0.0018	0.7401 ± 0.0178	0.6324 ± 0.0049	127.68 ± 0.9508
C	—	—	✓	3.01	8.1	0.9442 ± 0.0039	0.9532 ± 0.0090	0.9692 ± 0.0026	0.7453 ± 0.0107	0.6304 ± 0.0041	154.96 ± 0.7448
D	✓	—	✓	3.03	8.5	0.9467 ± 0.0029	0.9523 ± 0.0058	0.9721 ± 0.0019	0.7498 ± 0.0062	0.6349 ± 0.0024	127.27 ± 0.7101
E	✓	✓	—	2.62	7.5	0.9579 ± 0.0031	0.9586 ± 0.0026	0.9763 ± 0.0015	0.7431 ± 0.0152	0.6349 ± 0.0034	116.56 ± 0.8207
F	—	✓	✓	2.60	7.1	0.9527 ± 0.0159	0.9541 ± 0.0045	0.9758 ± 0.0019	0.7391 ± 0.0086	0.6300 ± 0.0038	116.80 ± 0.6702
G	✓	✓	✓	2.62	7.5	0.9766 ± 0.0048	0.9769 ± 0.0034	0.9888 ± 0.0009	0.8328 ± 0.0097	0.6894 ± 0.0024	155.78 ± 0.3940

P denotes Precision and R denotes Recall. Letters A–G represent different ablation experiment configurations, where “✓” indicates the inclusion of the corresponding module and “—" indicates its absence.

**Table 8:** Performance comparison before and after direct-wave removal.

Processing State	P	R	mAP@0.5	mAP@0.75	mAP@0.5:0.95
Original State	0.8688 ± 0.0133	0.9132 ± 0.0080	0.9524 ± 0.0045	0.7325 ± 0.0105	0.6199 ± 0.0048
After Average-trace Subtraction	0.9766 ± 0.0048	0.9769 ± 0.0034	0.9888 ± 0.0009	0.8328 ± 0.0097	0.6894 ± 0.0024

**Table 9:** Comparison of different feature extraction modules (Mean ± Std).

Model	Params	GFLOPs	P	R	mAP@0.5	mAP@0.75	mAP@0.5:0.95	FPS
HWD [28]	2.69	7.6	0.9476 ± 0.0096	0.9516 ± 0.0036	0.9693 ± 0.0005	0.7090 ± 0.0015	0.6230 ± 0.0116	98.87 ± 1.0453
SPD [29]	4.73	11.6	0.9536 ± 0.0047	0.9503 ± 0.0093	0.9696 ± 0.0011	0.7040 ± 0.0219	0.6215 ± 0.0062	47.81 ± 0.9056
Wavelet Pooling [30]	2.61	7.3	0.9420 ± 0.0033	0.9511 ± 0.0030	0.9693 ± 0.0013	0.7094 ± 0.0311	0.6186 ± 0.0067	97.46 ± 1.3151
PSConv	2.96	8.4	0.9506 ± 0.0041	0.9500 ± 0.0046	0.9724 ± 0.0015	0.7418 ± 0.0252	0.6321 ± 0.0044	104.79 ± 0.7400

**Table 10:** Performance comparison of different feature aggregation modules (Mean ± Std).

Model	Params	GFLOPs	P	R	mAP @0.5	mAP @0.75	mAP@0.5:0.95	FPS
CSP-EDLAN [31]	2.41	6.6	0.9354 ± 0.0109	0.9498 ± 0.0019	0.9609 ± 0.0067	0.7101 ± 0.0148	0.6193 ± 0.0051	97.95 ± 1.2478
RepNCSPELAN-DRB [32]	2.16	5.9	0.9438 ± 0.0025	0.9405 ± 0.0149	0.9644 ± 0.0094	0.7199 ± 0.0172	0.6219 ± 0.0021	86.57 ± 2.6903
RepNCSPELAN-OREPA [33]	2.19	5.9	0.9527 ± 0.0074	0.9491 ± 0.0082	0.9670 ± 0.0041	0.7256 ± 0.0054	0.6210 ± 0.0043	113.78 ± 1.0050
CSP-PMSFA	2.60	7.1	0.9488 ± 0.0011	0.9519 ± 0.0013	0.9726 ± 0.0018	0.7401 ± 0.0178	0.6324 ± 0.0049	127.68 ± 0.9508

**Table 11:** Comparative experimental results of different object detection algorithms over three random seeds (Mean ± Std).

Model	Params (M)	GFLOPs	mAP@0.5	mAP@0.75	mAP@0.5:0.95	FPS
YOLOv5n [34]	2.50	7.1	0.9752 ± 0.0017	0.7457 ± 0.0077	0.6321 ± 0.0038	148.35 ± 0.3387
YOLOv8n	3.01	8.1	0.9647 ± 0.0017	0.7069 ± 0.0240	0.6180 ± 0.0083	125.72 ± 0.0513
YOLOv10n [35]	2.27	6.5	0.9569 ± 0.0003	0.6997 ± 0.0124	0.6094 ± 0.0082	175.35 ± 0.6646
YOLOv11n	2.58	6.3	0.9744 ± 0.0033	0.7459 ± 0.0212	0.6325 ± 0.0044	105.95 ± 0.3536
YOLOv12n [36]	2.53	5.9	0.9700 ± 0.0009	0.7233 ± 0.0057	0.6281 ± 0.0030	104.75 ± 0.6439
Faster R-CNN	41.36	178	0.9586 ± 0.0025	0.6654 ± 0.0075	0.5880 ± 0.0026	11.00 ± 0.4583
TOOD [37]	32.02	168	0.9630 ± 0.0010	0.6827 ± 0.0098	0.6100 ± 0.0020	10.97 ± 0.7638
RTMDet-tiny [38]	4.87	8.0	0.9670 ± 0.0026	0.7340 ± 0.0053	0.6267 ± 0.0015	15.17 ± 0.4509
Ours	2.62	7.5	0.9888 ± 0.0009	0.8328 ± 0.0097	0.6894 ± 0.0024	155.78 ± 0.3940

**Table 12:** Generalization performance comparison between YOLOv8n and YOLOv8n-PCPG on the GPR Cavity dataset over three random seeds (0, 42, 1024) (Mean ± Std).

Model	P	R	mAP@0.5	mAP@0.75	mAP@0.5:0.95
YOLOv8n	0.9276 ± 0.0632	0.8907 ± 0.0412	0.9368 ± 0.0048	0.7745 ± 0.0424	0.6208 ± 0.0158
YOLOv8n-PCPG	0.9917 ± 0.0155	0.9402 ± 0.0102	0.9745 ± 0.0032	0.8175 ± 0.0297	0.6722 ± 0.0121

**Table 13:** Class-specific AP_0.5_ and overall mAP@0.5 performance comparison across target categories.

Model	Class Name(AP_0.5_)				mAP@0.5
	Pvc-Air	Pvc-Water	Pec	Void	Mean
YOLOv8n	0.957	0.955	0.966	0.976	0.963
YOLOv8n-PCPG	0.989	0.981	0.990	0.994	0.988

## Data Availability

Data underlying the results presented in this paper are not publicly available at this time but may be obtained from the authors upon reasonable request.

## Supplementary Materials

The following supporting information can be downloaded at: https://www.mdpi.com/article/10.3390/s26185872/s1, File S1: Python script for dataset generation using the gprMax API (uploaded as a .txt file).

## Abbreviations

The following abbreviations are used in this manuscript:

GPR	Ground-Penetrating Radar
YOLO	You Only Look Once
mAP	Mean Average Precision
PSConv	Pinwheel-Shaped Convolution
CSP-PMSFA	Cross-Stage Partial Multi-Scale Feature Aggregation
